# Newly-Appointed Consultants

**Published:** 1970-01

**Authors:** 


### NEWLY APPOINTED CONSULTANTS

### MR. L. R. CELESTIN

Mr. L. R. Celestin has been recently appointed Consultant Surgeon to the Departments of Gastro-enterology and of Surgery, Frenchay Hospital.

He was educated at the Royal College of Mauritius where he won a Gold Medal in Science and a Scholarship to study Medicine. After his studies at University College Hospital, London, he held several teaching Hospital appointments including a Registrarship and a Research Assistantship at the Royal Postgraduate Medical School, Hammersmith Hospital, before joining the B.R.I. as a Senior Registrar.

His publications have included articles on the œsophagus, gastro-intestinal hormones and herniation. He has lectured in the United States, Germany, France, Czechoslovakia and Portugal. His main gastro-enterological interests are with the stomach, colon and biliary system, while his general surgical ones lie with herniation and mammary lesions.

He now lives in Stoke Bishop.

### DR. R. LANGTON HEWER

Richard Langton Hewer has recently been appointed Consultant Neurologist in Bristol. Most of his work is being done at Frenchay Hospital, but he is also holding a Clinic at the Bristol Royal Infirmary. Dr. Hewer trained at St. Bartholomew's Hospital and subsequently held posts at the National Hospital for Nervous Diseases, Queen Square and in Oxford. He is especially interested in the rehabilitation aspect of neurology. He has also made a special study of Friedreich's Ataxia and allied disorders. He has three children and is living at Frenchay.

